# Observations on the Harveian Doctrine of the Circulation of the Blood

**Published:** 1818-05

**Authors:** 


*Observations on the Harveian Doctrine of the Circulation of the Blood.* By George Kerr. Small 8vo. pp. 100. London, 1816.

---

*Observations on the Harveian Doctrine of the Circulation of the Blood; in Reply to those lately adduced by George Kerr, Esq.* By Alexander Ewing, M. D. Member of the Royal Physical Society, Edinburgh. Small 8vo. pp. 251. London, 1817.

We are somewhat at a loss, whether we should apologise to our readers for not taking notice of Mr. Kerr's work sooner; or rather, whether we should not apologize to them for taking notice of it at all. It must be confessed, we were long doubtful as to the propriety of doing the latter, and have been swayed to it only by the consideration that, in presenting a graphic representation of the passing current of Medical Literature, it becomes the duty of our office, not only to describe the depth of the flood, and the direction of the main-stream, but also to take occasional notice of the off-sets or eddies that whimsically play about its edges, and those frothy bubbles that rise to whirl, and burst, and sink again, on its rippling surface. Besides, we conceive our readers cannot fail to regard as a real curiosity, a work written, in the Year of our Lord One Thousand Eight Hundred and Sixteen, professedly against the circulation of the blood! In addition to all this, it is not impossible, but that mere capriciousness of taste has had its share in leading us to fasten upon this singular production; and that, feeling (like the French Abbé) the loathsomeness of "toujours perdrix," we have been induced, for a short season, to forsake nobler literary game, and—we will not say "to prey on garbage"—but, to sate the keenness of our critical appetite, on

"Rats and mice, and such small deer."*

Let not Mr. Kerr take offence at being likened unto animals so ignoble: we wish the similitude to be understood as merely a literary one; and we employ it only with a reference to his nibbling at established doctrines, and his present attempt to *eat through* the *bottom plank* in the theory and practice of our art—namely, the Harveian discovery of the circulation.

As to that attempt, we are constrained to say, that it is almost as feeble in its execution, as it is hopeless in its result; and that the author, with all his scholarship and all his verbiage (for we do not deny him a certain species of talent), has evinced reasoning powers of the very lowest order, and a bigotted attachment to the reveries of the ancients, worthy of the dark ages. Since the time that the Aristotelian philosophy was overthrown, and that Bacon, Harvey, Boyle, and Newton taught the true method of philosophising—as well by their weighty precepts as by their mighty example—we did not conceive it possible for any one to be hardy enough to advocate seriously the absurd speculations of the ancients in physical and experimental science. But Mr. Kerr has outgone our expectations, and has produced a work, we will venture to say, perfectly unique in its kind. Though not without plausibility, and not without expertness in laying hold of so much as makes for his purpose and no more, he seems to be imbued with so thorough a persuasion of his own superior sagacity, that he has been constantly led into the error of talking big, and of substituting vociferation for ratiocination, and emphasis for argument. Hence his hundred pages, bedecked though they be with here and there a tidy Greek quotation, and redeemed as they are from absolute contempt, by a thin coating of ingenuity,—may be fairly characterised in the sententious, but expressive language of the philosophic Sallust, as containing "satis loquentiæ, sapientiæ parum."

* Shakspeare's King Lear.

The opinions of this modern Riolanus are, for the most part, undeserving of sober reply; and as for his arguments, if such indeed they can be called, they seem chiefly to belong to that peculiar class to which our Uncle Toby, of respected memory, would have replied, by whistling Lillabullero,—that is to say, if that worthy man had been as intimately acquainted with the moving power of the heart and the impulse of the blood, as he confessedly was with some of the other branches of projectiles!

Let us now look into this outrè production.—After a Preface of twenty-one pages, which may be said to be "de omni scibili,"—and of which, by consequence, it would be very difficult to give any clear or reasonable account; the author commences his disquisition in the following terms:

"It has often appeared to me very extraordinary, that while we admit the superiority of the ancients in poetry, history, oratory, mathematics; and hold as models their architecture, statuary, engravings, and all works of art that have been preserved to our times, we should yet consider their medical and chirurgical works as unworthy of our regard—although we have the most unquestionable proofs, that many of the physicians of antiquity were men of the first talents, and these talents improved by unwearied attention to professional studies." P. 1.

Now this passage is highly sophistical, and we shall take some pains to refute it, as by so doing, we shall go nigh to invalidate all the arguments of a similar stamp, which this unreasonable "laudator temporis acti" advances in the sequel.

The author here proceeds upon an assumed analogy, that as the ancients excelled in poetry and the fine arts, they must needs, by parity of reason, excel as much in the more difficult branches of experimental study. This is the precise amount and weight of his argument; and it is sufficient to convince us, that of all other modes of reasoning, analogy is the one most liable to be abused. Like an edge-tool, it is a most dangerous weapon in unskilful hands; and the use which Mr. Kerr has here made of it, sanctions us in saying so. We must make free to tell him that the analogy he takes for granted is a false one, and has no existence; for we contend, that there is a wide difference between the imitative arts, and the higher branches of moral or physical science; and that the latter require not only severer habits of study, but even powers of mind different from the former. It is possible, for instance, for a man to possess exquisite taste in architecture, sculpture, or painting, and at the same time, to be a very indifferent reasoner; and it is equally possible—for it is what we have all seen now and then—that an individual shall possess great expanse of imagination, and a power of fancy sufficient to embody thought in the most glowing and animated poetical or rhetorical diction, without necessarily possessing, at the same time, that cautious solidity of judgment, and that patience of investigation, requisite for the pursuit and discovery of moral or experimental truth.

Indeed, it appears to us, that skill in the fine arts is the result of a special power—an intuitive faculty of imitation, which experience has proved to be neither referable to, nor connected with, strength, or even with culture of the understanding.

Let it not be said, that poetry and oratory form an objection to this reasoning. As for the former, Aristotle himself has called it an imitative art, and the essence of both we believe to be a susceptibility of strong emotion, and a power of imitating or pourtraying that emotion, so as to produce impressions of joy or sorrow, gaiety or sublimity, terror or tenderness, in the minds of others. Hence we are disposed to place them on the same level with the other arts more strictly imitative, and to consign them over to the province of taste rather than to that of intellect. That no analogical inference can be drawn from the pitch of perfection to which they may be carried, is sufficiently manifest from the well-known fact, that both poetry and oratory have been found to flourish in their greatest vigour in the earliest ages, and in states of society the very reverse of enlightened. Nay, examples might be cited from the writings of Homer or of Ossian, and from the harangues of the Indian Chiefs in the wilds of America, which, for energy of conception, boldness of personification, scope of metaphor, and refinement of tenderness, stand almost (and we need scarcely say *almost)* unrivalled through succeeding ages. In fact, strong emotion, which, as we said before, is the very *pabulum* of the poetical and rhetorical fire, seems peculiarly the offspring of imperfect civilization, seeing it is constantly roused by the external circumstances and transitions, the hopes, the wants, the fears, the joys, or the sufferings of savage life. Hence it is, that the earlier poets, orators, or sculptors, by laying hold of the grander images, and the more prominent features of external nature or of human character, have carried their respective arts to a high state of perfection, and have acquired the character of being at once more powerful and more natural than most of their modern successors, who, though they may have the same capacities, have not the same possibilities of excellence.

But the case is far different in moral and mechanical philosophy. In the latter particularly, as Bacon has well remarked, truth is the daughter of time, and not of imitation or intuition. From the very nature of things, a lapse of ages seems absolutely necessary, not merely for instituting experiments and making discoveries, but for the very preparation and culture of the mind requisite to success. It is not, as in the fine arts, by the occasional out-breakings of individual genius, or the fortuitous coruscations of ingenuity, that proficiency is here to be acquired. On the contrary, it may very fairly be debated, that these have been of infinite prejudice in every part of experimental science, and more particularly in medicine; simply from their profusely generating hypotheses, which, like the idols of old, have seduced men away from the true altar of fact and observation. In our own profession, we begin to be tired of the *Ariels*—the wonder-working spirits that figure in the etherial regions of speculation, comely although they be,—and are almost inclined to go into the opinion of M. de Buffon, that "genius is only an aptitude to patience," and that *it* is the sort of genius which is calculated, on the long run, to do most good.

These considerations, hastily and imperfectly as they are traced, will sufficiently explain the fact—a fact confirmed by the clearest testimony of history, that the ancients, with all their genius and all their eminence in the Belles Lettres, were comparatively mere drivellers in the philosophy either of mind or matter; and that their opinions, from the mystical speculations of Plato and Antoninus, down to the grosser dogmas of Pyrrho or Epicurus, and from the long reverenced tenets of Aristotle to the now incredible reveries of Galen, Erasistratus or Pliny, exhibited little else than a picture of the imbecility and the caprice of the human mind, and have no claim to attention at this advanced æra of knowledge.

We would ask Mr. Kerr, then, why we must still bend the knee at the footstool of antiquity? Are they wiser than we, simply from having lived before us? "Non enim (says the eloquent Lactantius *) quia nos illi tem-

* Lactant. Div. Institut. lib. ii. cap. 8.

poribus antecesserunt, sapientiâ quoque antecesserunt." Indeed, the very reverse is the case, as has been well explained by one of the most distinguished philosophers of the present age, in the following passage, which we quote not merely as making to our purpose, but with a view of recommending the whole of his admirable dissertation to the perusal of our readers.

* "One ought to listen with caution to the encomiums sometimes bestowed on the philosophy of those early ages. If these encomiums respected only the genius, the taste, the talents, of the great masters of antiquity, we would subscribe to them without any apprehension of going beyond the truth. But if they extend to the methods of philosophising and the discoveries actually made, we must be excused for entering our dissent, and exchanging the language of panegyric for that of apology. The infancy of science could not be the time when its attainments were the highest; and before we suffer ourselves to be guided by the veneration of antiquity, we ought to consider *in what real antiquity* consists. With regard to the progress of knowledge and improvement, 'we are more ancient than those who went before us.†' The human race has now more experience than in the generations that are past, and of course may have been expected to have made higher attainments in science and philosophy. Compared with natural philosophy as it now exists, the ancient physics were rude and imperfect. Truth and falsehood met almost in terms of equality; the former, separated from its root experience, found no preference above the latter; to the latter, in fact, it was generally forced to give way, and the dominion of error was finally established."

Mr. Kerr's objections against Harvey's doctrine of the circulation, are no less than nine-fold, and are summarily these:—1st. That the heart continues its alternate contractions and relaxations for some time after it is cut out of the body, when it has obviously no blood to circulate. 2d. The pulse cannot be an "impulsus sanguinis in arteriis," because it is transmitted instantaneously. 3d. When an artery is wounded, the blood appears venous, and pulsation is lost. 4th. The empty state of the arteries after death. 5th. Cases of phthisis, where one lobe of the lungs is wholly destroyed, without any consequent effusion of blood. 6th. The quantity of blood in

---

* Dissertation on the Progress of Mathematical and Physical Science, by Professor Playfair, of Edinburgh, prefixed to the Supplement to the Encyclopædia Britannica, 1817.

† Lord Bacon.

the body is not sufficient to fill both arteries and veins at the same time. 7th. The ligature of the saphena vein occasions no swelling of the foot. 8th. The arteries are found empty after death, even though violent exercise, or heat, may have caused turgidity of the superficial vessels. 9th. The valves in the veins do not prove the circulation, because there are also valves in the lymphatics, though in the latter no circulation exists.

To these puerile objections, some of which are false, and the others easily explicable on the well ascertained laws of the sanguiferous system, Dr. Ewing has thought proper to direct a laboured methodical reply of two hundred and fifty-one pages! He displays a great deal of research, and no inconsiderable share of learning; so much so, that wherever his adversary thunders in Greek, he returns him broadside for broadside in the same language; and when he takes shelter under the wing of Erasistratus or Galen, or Paulus Ægineta, the other is sure to pluck the feathers from his place of refuge. We have no fault to find with what Dr. Ewing has written: on the contrary, we can recommend his arguments as being quite conclusive; but indeed they could hardly be otherwise with such an easy cause to defend. We would engage simply, from Harvey's "*Exercitatio Anatomica de motu Cordis*" (putting all modern works out of the question), to produce incontestible arguments for the circulation. In short, we consider that it is indubitably proved by *one* fact, namely, the existence of valves in the veins and in the heart. As Nature makes nothing in vain, these must have the same use as similar contrivances in hydraulic machines. The argument deduced from the consideration of final causes is highly conclusive; and the evident intention of a peculiar structure, in a case of this kind (like a matter of fact in ordinary life), cannot be annulled or impugned by a volume of sophistical doubts or subtle objections. This, we think, is all that needed to have been said on the subject; and we cannot help thinking that Dr. E. has spent his time very unsuccessfully in honouring such absurdities with a lengthened answer. Who would bring up a mortar to demolish a mole-hill, or gird on his armour to put down a man of straw?

Mark, reader, the luminous, the philosophic, the satisfactory hypothesis which Mr. Kerr brings forward, after laying the Harveian doctrine in the dust!

"Some years ago Mr. Wilkinson, of London, published 'Elements of Galvanism,' in which he introduced an hypothesis, which to me appeared probable and very ingenious; viz. 'That the lungs collect elemental fire from the atmosphere; that the bronchiæ, resembling in structure the cells of the torpedo, possess an action somewhat similar; and that the *pulse* is literally a shock sent through the arteries.' I thought this hypothesis probable, because the pulse is transmitted instantaneously, and not, according to the law of fluids, propelled through elastic tubes: because pure air is known to contain elemental fire, as we inhale it; and in expiration is found to have lost it: and because, in cases of increased heat of any part of the body, attended with pain, the most immediate relief is obtained by the application of cold water, or the contact of cold metals, our most perfect conductors." P. 80.

This then, it seems, is the famous succedaneum for the circulation of the blood, the recipe for which our author first got from Mr. Wilkinson, and which he is now vending under the old-established firm of Messrs. Erasistratus, Kerr, and Co. We do not pretend to understand all its merits, but we can honestly say, that it far surpasses any theory ever before offered to the medical world. In fact, it out-herods Herod; and after it the dreams of Swedenborg, or the lucubrations of Dr. Faustus, may hide their diminished heads. We have always conceived, that whosoever would patch up a right-witty and fanciful hypothesis, it is of all things necessary that he should first varnish it over with some resemblance, however faint, to plausibility, if not to probability, and should get, by some means or other, two or three solitary facts, to give it a shew of support. But now-a-days, it seems, this is not at all necessary, for, in the present instance, the hypothesis is advanced not only without facts, but in the very face of facts, and claims to itself the distinction of being the most gratuitous theory that we ever remember to have heard or read of. Nevertheless, we can see that it is most firmly believed, with all its faults — we mean by Mr. Kerr himself; but as to what degree of success he may have in making proselytes, it passeth our skill to say. Let him not despair, however: Joanna Southcot gained many converts, though her cause seemed, à priori, absolutely hopeless.

We solicit our readers' attention to the hardihood of the following assertion, satisfied that they will agree with us, in thinking that a work which contains such sentiments, not only justifies but calls for memorable severity of criticism.

"It is matter of notoriety, that Harvey's discovery of the circulation has not proved useful, and has not simplified medical practice, or afforded better means of relieving disease." P. 11—78.

This is merely one instance of the author's unreasonableness; but throughout the whole performance he deals largely in round assertions, and efforts at argumentation, which expose only his own impotency. His fruitless conflict with the opinions of Harvey has something so ludicrous in it, as to remind us of the homely picture drawn by the humorous Muse of Dan Prior:

"Dear Thomas, didst thou never pop
Thy head into a Tin-man's shop?
There, Thomas, didst thou never see
('Tis but by way of simile)
A Squirrel spend his little rage
In jumping round a rolling cage?
—The cage, as either side turns up,
Striking a ring of bells a-top:
Mov'd in the orb, pleas'd with the chimes,
The hapless creature thinks he climbs;
But here or there, turn wood or wire,
He *never gets two inches higher!*"

Now, were Mr. Kerr merely unreasonable, we should feel a pleasure in pitying him, and opening his eyes to better views: but he is arrogant as well; and at every turn irritates us by his dogmatism. He seems to entertain a very favourable opinion of his own powers, and is at no loss to speak in praise of himself. The latter happy qualification, we presume, he has copied from his beloved ancients, who excelled in it to a pre-eminent degree. Almost to a man they are egotistical, and insensible to the indelicacy of speaking in their own praise. Cicero omits no opportunity of lavishing encomiums on his own merits; and even Seneca—the sensible, the philosophic Seneca—takes occasion, in his dying words, to pass upon himself a compliment, which any modern (save, perhaps, the modern Riolanus) would blush to utter.—"But this eternal blazon must not be," and we would recommend Mr. Kerr to drop the custom, and, for some time to come, to direct all his skill to repress those troublesome and unseemly tumours which spring from the broad radicle of his personal vanity. Lest he should not have courage to become his own physician, we have taken the liberty to apply a blister to the morbid parts, made up of caustic criticism.

By the bye, as our author is so fond of Greek, we beg leave to give him, at parting, the following short quotation, leaving him to digest it at his leisure, and, if he thinks fit, to "translate it (as the celebrated Mr. Sheridan was wont facetiously to say), for the benefit of the country gentlemen:"

